# 16S rRNA Gene Amplicon Sequencing of Gut Microbiota in Three Species of Deep-Sea Fish in Suruga Bay, Japan

**DOI:** 10.1128/MRA.01260-20

**Published:** 2021-01-07

**Authors:** Toshihide Iwatsuki, Takahiro Kanazawa, Takato Ogasawara, Kento Hosotani, Karen Tsuchiya, Shinichi Watanabe, Tomoko Suzuki, Ryota Moriuchi, Yu Kanesaki, Hideo Dohra

**Affiliations:** a Numazu Higashi High School, Numazu, Japan; b Research Institute of Green Science and Technology, Shizuoka University, Shizuoka, Japan; c Department of Science, Graduate School of Integrated Science and Technology, Shizuoka University, Shizuoka, Japan; Indiana University, Bloomington

## Abstract

We report here 16S rRNA gene amplicon sequence analysis of the gut microbiota in three species of deep-sea fish collected from Suruga Bay, Japan. Of the three species, two were dominated by the phylum *Proteobacteria* (genus *Photobacterium*), while one was dominated by the phyla *Spirochaetes* (genus *Brevinema*) and *Tenericutes* (unclassified *Mycoplasmataceae*).

## ANNOUNCEMENT

The gut microbiota in fish plays a critical role in host physiology (1, 2) and is affected by many factors, such as diet, environment, and host phylogeny (3–6). However, information on the gut microbiota of deep-sea fish is still limited (7, 8). In this study, we analyzed the gut microbiota of fish collected from the deep sea, which has a unique environment with low biodiversity, high pressure, and low water temperature.

Three species of deep-sea fish (Chlorophthalmus albatrossis, Glossanodon semifasciatus, and Helicolenus hilgendorfi) were caught with a trawl net at a depth of approximately 300 m in Suruga Bay, Japan (34.575N, 138.710E). Their gut contents were collected with dissection and stored at −80°C until use. Total DNA was extracted from the gut contents (150 to 200 mg) of three different fish of the same species of deep-sea fish using the DNeasy PowerSoil kit (Qiagen) following homogenization with a personal Minilys homogenizer (Bertin Instruments) at 5,000 rpm for 10 min. The V3-V4 region of the 16S rRNA gene was amplified using primers 341F (5′-ACACTCTTTCCCTACACGACGCTCTTCCGATCT-NNNNN-CCTACGGGNGGCWGCAG-3′) and 805R (5′-GTGACTGGAGTTCAGACGTGTGCTCTTCCGATCT-NNNNN-GACTACHVGGGTATCTAATCC-3′) and MightyAmp DNA polymerase v.3 (TaKaRa Bio, Inc.) according to the manufacturer’s instructions except for annealing at 55°C. Library construction and sequencing were performed at the Bioengineering Lab Co., Ltd. The libraries were sequenced using a MiSeq platform (Illumina) to generate 301-bp paired-end reads. The sequence data were analyzed using EzBioCloud 16S rRNA gene-based microbiome taxonomic profiling (MTP) (ChunLab, Inc.) (9) with the following parameters: “Bacteria” as a target taxon and the prokaryotic 16S rRNA gene database PKSSU4.0. Sequences processed in the EzBioCloud 16S rRNA gene-based MTP pipeline were subjected to taxonomic assignment using the sequence identity thresholds proposed previously (10). Sample and sequence data are summarized in Table 1.

**TABLE 1:** Summary of samples analyzed in this study

Sample name	Host species	Collection date (yr-mo-day)	No. of raw reads	No. of filtered reads	No. of valid reads^*a*^	% of valid reads	Accession no.
Ca_1	*Chlorophthalmus albatrossis*	2019-09-29	47,572	44,146	43,527	98.6	DRR237428
Ca_2	*Chlorophthalmus albatrossis*	2019-09-29	48,528	45,115	43,362	96.1	DRR237429
Ca_3	*Chlorophthalmus albatrossis*	2019-09-29	39,556	37,061	18,389	49.6^*b*^	DRR237430
Hh_1	*Helicolenus hilgendorfi*	2019-09-29	36,923	34,085	32,609	95.7	DRR237431
Hh_2	*Helicolenus hilgendorfi*	2019-09-29	46,178	42,745	41,997	98.3	DRR237432
Hh_3	*Helicolenus hilgendorfi*	2019-09-29	30,911	28,374	23,365	82.3	DRR237433
Gs_1	*Glossanodon semifasciatus*	2019-11-16	39,601	37,264	36,756	98.6	DRR237434
Gs_2	*Glossanodon semifasciatus*	2019-11-16	49,989	46,936	46,066	98.1	DRR237435
Gs_3	*Glossanodon semifasciatus*	2019-11-16	37,283	34,499	33,848	98.1	DRR237436

a Valid reads indicate the reads excluding low-quality (averaged quality value of <25), nontarget, and chimeric reads processed in the EzBioCloud 16S rRNA gene-based MTP pipeline, which were used for the microbiota taxonomic analyses.

b The low percentage of valid reads for sample Ca_3 is due to amplification of the organelle rRNA gene in eukaryotic parasites.

**FIG 1:**
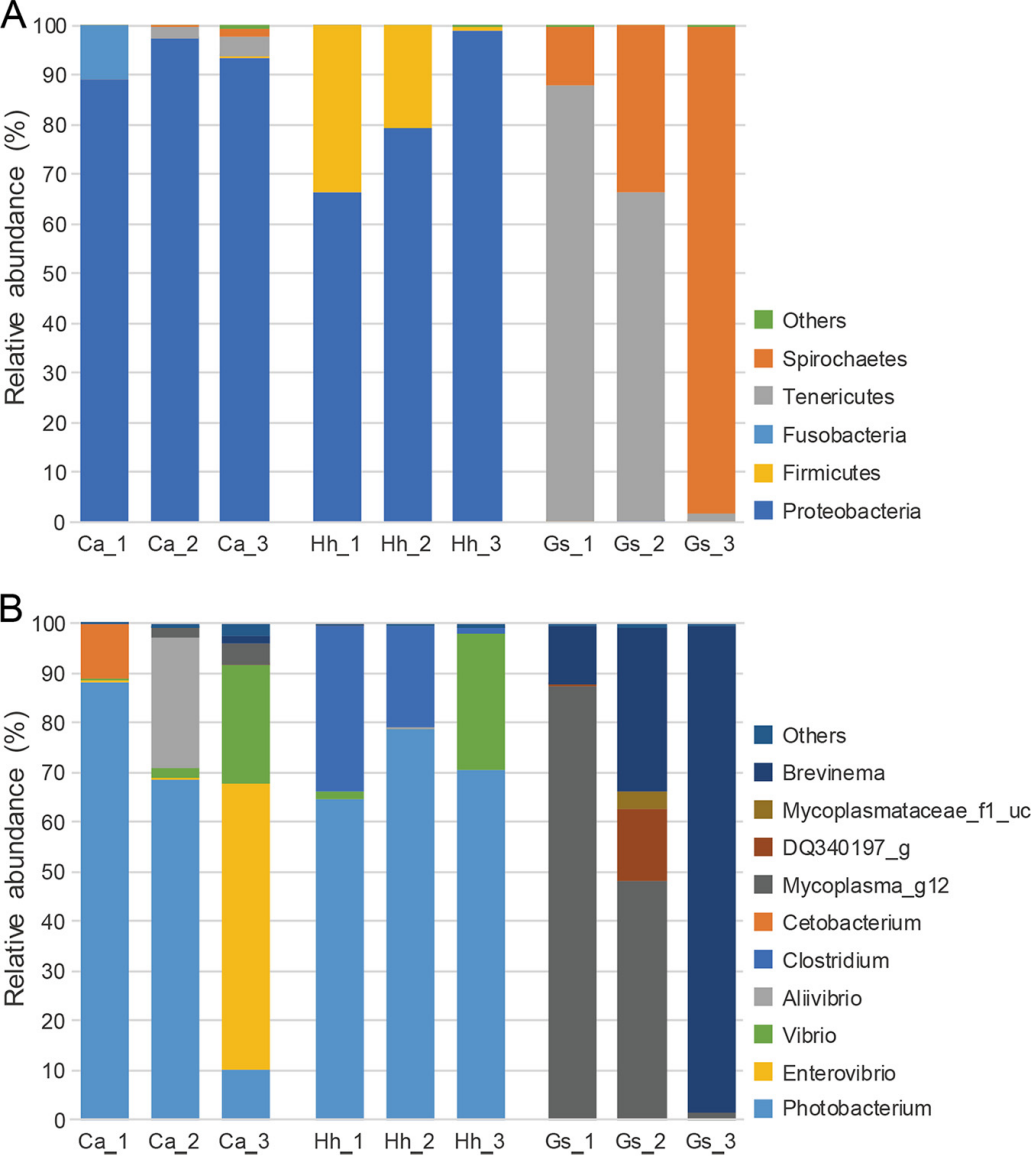
Bar chart representing the taxonomic composition of the gut microbiota in deep-sea fish, based on 16S rRNA gene amplicon sequence analysis. Relative abundances of taxa are shown at the phylum level (A) and the genus level (B). Sample names indicate host species of *Chlorophthalmus albatrossis* (Ca), *Helicolenus hilgendorfi* (Hh), and *Glossanodon semifasciatus* (Gs). Others include taxa with a relative abundance of less than 1%. DQ340197_g in panel B is an uncultured *Mycoplasma* sp. belonging to the family *Mycoplasmataceae* reported in the gut microbiota of a long-jawed mudsucker (12).

In the gut microbiota of C. albatrossis and H. hilgendorfi, the phylum *Proteobacteria* dominated, with relative abundances of 66.4 to 98.7% (Fig. 1A). The most dominant genus was *Photobacterium* (*Proteobacteria*), followed by *Vibrio*, *Enterovibrio*, *Aliivibrio* (*Proteobacteria*), and *Clostridium* (*Firmicutes*) (Fig. 1B). *Photobacterium* spp. are also known as symbiotic luminous bacteria in light organs of C. albatrossis (11). These trends were well in accordance with a previous review in which the dominant genera of the fish gut microbiota from over 30 studies were *Vibrio*, *Photobacterium*, and *Clostridium* (5), suggesting that the taxonomic composition of the gut microbiota in C. albatrossis and H. hilgendorfi is not unique to deep-sea fish. On the other hand, the gut microbiota of G. semifasciatus was dominated by the phyla *Spirochaetes* (genus *Brevinema*) and *Tenericutes* (unclassified *Mycoplasmataceae*) (Fig. 1A and B). The dominance of *Mycoplasma* was also reported for the gut microbiota of a long-jawed mudsucker from salt marshes in California (12). In all samples, the top one to four genera accounted for more than 95% of the gut microbiota (Fig. 1B), suggesting that deep-sea fish have a low-diversity gut microbiota.

### Data availability.

The 16S rRNA gene amplicon sequence data have been deposited in the DDBJ Sequence Read Archive (DRA) under the accession numbers DRR237428 to DRR237436.
